# Radiotherapy with or without Decompressive Surgery for Metastatic Spinal Cord Compression: A Retrospective Matched-Pair Study Including Data from Prospectively Evaluated Patients

**DOI:** 10.3390/cancers14051260

**Published:** 2022-02-28

**Authors:** Dirk Rades, Jan Küchler, Lena Graumüller, Abdulkareem Abusamha, Steven E. Schild, Jan Gliemroth

**Affiliations:** 1 Department of Radiation Oncology, University of Lubeck, 23562 Lubeck, Germany; 2 Department of Neurosurgery, University of Lubeck, 23562 Lubeck, Germany; lena.graumueller@student.uni-luebeck.de (L.G.); abdulkareem.abusamha@uksh.de (A.A.); jan.gliemroth@uksh.de (J.G.); 3 Department of Radiation Oncology, Mayo Clinic, Scottsdale, AZ 85259, USA; sschild@mayo.edu

**Keywords:** metastatic spinal cord compression, radiotherapy, decompressive surgery, motor function, local control

## Abstract

**Simple Summary:**

In a retrospective matched-pair study including data of prospectively evaluated patients who were treated for metastatic spinal cord compression, 79 patients assigned to surgery plus radiotherapy were compared to 79 patients receiving radiotherapy alone. Improvement of motor function occurred more significantly often after surgery plus radiotherapy, whereas no significant differences were found for post-treatment ambulatory rates, local progression-free survival, overall survival, and freedom from in-field recurrence. Ten patients died within 30 days after radiotherapy alone and 12 patients within 30 days after surgery. More than one third of surgically treated patients did not complete their radiotherapy due to early death or decreased performance score following surgery. Thus, when selecting a patient for upfront surgery, the individual patient’s prognosis must be considered and weighed against the risk of perioperative complications and 30-day mortality.

**Abstract:**

In 2005, a randomized trial showed that addition of surgery to radiotherapy improved outcomes in patients with metastatic spinal cord compression (MSCC). Since then, only a few studies compared radiotherapy plus surgery to radiotherapy alone. We performed a retrospective matched-pair study including data from prospective cohorts treated after 2005. Seventy-nine patients receiving radiotherapy alone were matched to 79 patients assigned to surgery plus radiotherapy (propensity score method) for age, gender, performance score, tumor type, affected vertebrae, other bone or visceral metastases, interval tumor diagnosis to MSCC, time developing motor deficits, and ambulatory status. Improvement of motor function by ≥1 Frankel grade occurred more often after surgery plus radiotherapy (39.2% vs. 21.5%, *p* = 0.015). No significant differences were found for post-treatment ambulatory rates (59.5% vs. 67.1%, *p* = 0.32), local progression-free survival (*p* = 0.47), overall survival (*p* = 0.51), and freedom from in-field recurrence of MSCC (90.1% vs. 76.2% at 12 months, *p* = 0.58). Ten patients (12.7%) died within 30 days following radiotherapy alone and 12 patients (15.2%) died within 30 days following surgery (*p* = 0.65); 36.7% of surgically treated patients did not complete radiotherapy as planned. Surgery led to significant early improvement of motor function and non-significantly better long-term control. Patients scheduled for surgery must be carefully selected considering potential benefits and risk of perioperative complications.

## 1. Introduction

Metastatic spinal cord compression (MSCC) is considered an oncologic emergency and occurs, depending on the primary tumor type, in up to 10% of patients with cancer or malignant hematological disease [1,2,3]. The optimum treatment for MSCC is still controversial. Radiotherapy alone had been the standard treatment for decades. In 2005, a randomized trial showed that the addition of upfront decompressive surgery to radiotherapy was associated with improved outcomes, including post-treatment ambulatory status and survival in selected patients with a good performance status, an expected survival of at least 3 months, involvement of a single area, and paraplegia for no longer than 48 h [4]. Moreover, patients with very radiosensitive tumors, such as myeloma, lymphoma, and germ-cell tumors, were not included. Since this trial was published, the combined approach has become considerably more popular [4]. Despite the fact that the trial was criticized because of significant methodological problems, no subsequent randomized trials were performed. Since 2005, only a few (non-randomized) studies have compared radiotherapy plus upfront surgery and radiotherapy alone. In 2010, a retrospective matched-pair analysis in a less selected cohort of 324 patients mainly treated before 2006 was presented [5]. One-hundred-and-eight patients receiving radiotherapy plus surgery were matched 1:2 to 216 patients receiving radiotherapy alone considering 11 potential prognostic factors. In this study post-treatment outcomes were not significantly different. In an additional retrospective matched-pair study of 201 patients with MSCC from an unfavorable primary tumor matched 1:2 (similar approach as in the previous study), post-treatment outcomes were also not significantly different [6]. However, in a subgroup analysis of the 129 (43 vs. 86) patients receiving decompressive surgery plus stabilization, the addition of upfront surgery was associated with a higher rate of improvement of motor deficits (28% vs. 19%, *p* = 0.024). In a small retrospective cohort study (*n* = 88) reported in 2019, aggravation of impending paralysis was observed in 16.7% of 18 patients receiving decompression and stabilization, in 13.3% of 15 patients receiving stabilization without decompression and in 16.7% of 55 patients treated with radiotherapy [7]. However, these three studies were limited by their retrospective designs [5,6,7]. Considering the low number of studies comparing surgery plus radiotherapy and radiotherapy alone for MSCC and their significant limitations, it becomes obvious that additional studies are required. Moreover, techniques of both surgery and radiotherapy have significantly improved since 2005 [8,9,10].

However, as mentioned above, another randomized trial is difficult to perform, since treating physicians are often hesitant to omit upfront surgery. Therefore, we performed a new matched-pair study, including patients from four prospective radiotherapy trials, and a validation study [9,10,11,12,13], and from a large prospectively developed database of neurosurgical patients.

## 2. Results

After final propensity-score matching, 79 patients receiving radiotherapy alone remained for comparisons with the cohort of 79 patients assigned to surgery plus radiotherapy. In the radiotherapy alone group, all 79 patients completed their radiotherapy as planned with 5 × 5 Gy (*n* = 8). 10 × 3 Gy (*n* = 55), 14−15 × 2.5 Gy (*n* = 5), 15 × 2.633 Gy (*n* = 4) or 20 × 2 Gy (*n* = 7). In the surgery plus radiotherapy group, 50 patients received post-operative irradiation with an equivalent dose in 2 Gy fractions (EQD2, [14]) higher than 30 Gy, i.e., with 5 × 5 Gy (*n* = 1), 10−11 × 3 Gy (*n* = 27), 13−15 × 2.5 Gy (*n* = 13), or 18−20 × 2 Gy (*n* = 9), respectively. Twelve patients (15.2%) did not receive any radiotherapy, because they died after surgery before the planned post-operative irradiation was started. Causes of death included acute respiratory failure/pneumonia (*n* = 5), sepsis (*n* = 1), acute liver failure (*n* = 1), rapid tumor progression (*n* = 1), ileus (*n* = 1), intracranial hemorrhage (*n* = 1), and pulmonary embolism (*n* = 1). Cause of death remained unclear in one patient. Moreover, 17 patients (21.5%) did not receive the radiotherapy as planned due a decreased performance score following surgery. Eleven of these patients received short-course radiotherapy with 5 × 4 Gy (*n* = 10) or 6 × 4 Gy (*n* = 1) instead of planned longer-course radiotherapy. In three patients, radiotherapy was discontinued after 5 × 3 Gy (of 10 × 3 Gy), 7 × 3 Gy (of 10 × 3 Gy) and 11 × 2 Gy (of 20 × 2 Gy), respectively. Another three patients received only one fraction of 2 Gy, 2.5 Gy or 3 Gy, respectively.

In the surgery plus radiotherapy group, the 29 patients who did not complete their radiotherapy as planned had a significantly worse Eastern Cooperative Oncology Group performance score (ECOG-PS) and were significantly more often not ambulatory than the 50 patients who completed post-operative irradiation (Table 1). Otherwise, the patient characteristics were not significantly different between these groups. In the entire cohort, median follow-up times were 4 months for all patients and 10 months for those who were alive at the last follow-up visit. Follow-up times were 3 and 9 months in the surgery plus radiotherapy group, and 6 and 12 months in the radiotherapy alone group, respectively.

The comparisons of both groups for the investigated endpoints showed improvement of motor function to be achieved significantly more often after surgery plus radiotherapy than after radiotherapy alone (39.2% vs. 21.5%, *p* = 0.015). In the subsequent multivariate analysis (after stepwise regression modeling), surgery plus radiotherapy maintained significance (odds ratio 2.55; 95% confidence interval 1.25–5.22, *p* = 0.011). Non-significantly more patients in the surgery plus radiotherapy experienced deterioration of motor deficits (15.2% vs. 8.9%, *p* = 0.22). Of these 12 patients, 11 had an interval from tumor diagnosis to MSCC ≤15 months, 8 had an ECOG-PS of 3–4, and there were 7 visceral metastases. When comparing the seven patients with all three of these characteristics to the other 72 patients in the surgery plus radiotherapy group, the rates of deterioration of motor deficits were 71.4% and 9.7%, respectively (*p* < 0.001, Fisher’s exact test).

No significant differences between radiotherapy plus surgery and radiotherapy alone were found for post-treatment ambulatory rates (59.5% vs. 67.1%, *p* = 0.32), local progression-free survival (*p* = 0.47), overall survival (*p* = 0.51), and freedom from an in-field recurrence of MSCC with new or progressive motor deficits following treatment (*p* = 0.58). Median overall survival times were 7 months and 7 months, respectively. Ten patients (12.7%) died within 30 days following radiotherapy alone, and 12 patients (15.2%) within 30 days following surgery in the surgery plus radiotherapy group (*p* = 0.65). The results with respect to all investigated endpoints are shown in Table 2 and Table 3.

## 3. Discussion

Until 2005, radiotherapy alone had been the designated standard treatment for MSCC. However, in that year, a randomized trial was published that compared 10 × 3 Gy of radiotherapy alone to the same regimen plus upfront decompressive surgery [4]. The results of this trial were in favor of the combined treatment. Post-treatment ambulatory rates were 84% (42 of 50 patients) and 57% (29 of 51 patients), respectively (*p* = 0.001). Moreover, patients of the surgery plus radiotherapy group maintained their gait function significantly longer (median 122 vs. 13 days, *p* = 0.003) and had a significantly better survival (median 4.2 vs. 3.3 months, *p* = 0.033). However, this trial was criticized for significant limitations [15,16,17]. Since it took 10 years to include 101 patients, these appear highly selected. Thirty-eight patients (18 in the radiotherapy alone group) had an unstable spine, which is a clear indication for surgery and, therefore, likely led to a bias in favor of the combined treatment. This may explain why the results after radiotherapy alone were considerably worse than in most other studies. Although this trial suffered from major limitations, an additional randomized trial comparing surgery plus radiotherapy and radiotherapy alone has not been performed since and is not expected in the near future. If a randomized trial cannot be performed, a matched-pair study following strict criteria may provide the highest possible level of evidence. Such a study was reported in 2010 [5]. It included a larger number of patients (*n* = 324, matched 1:2 for 11 clinical and demographic variables) than the Patchell trial and excluded patients with vertebral fractures and bony fragments in the spinal canal, since these situations usually cannot be treated successfully with radiotherapy alone [1,2,3]. The patients of that matched-pair study were less selected and also included very radiosensitive tumors [5]. No significant differences were found regarding improvement of motor function, post-treatment ambulatory rates, local control rates of MSCC at 1 year, and 1-year survival rates. It was concluded that the outcomes after surgery plus radiotherapy and radiotherapy alone appeared similar [5]. In order to identify groups of patients who could benefit from upfront surgery, a subsequent matched-pair study was performed in patients with unfavorable and less radiosensitive tumors, namely non-small cell lung cancer, cancer of unknown primary, renal cell carcinoma and colorectal cancers [6]. Both treatments were similarly effective with respect to all but one investigated endpoint. Improvement of motor function was observed significantly more often in the subgroup of patients who received decompressive surgery plus stabilization (28% vs. 19%, *p* = 0.024).

When looking at the data of these studies, it becomes obvious that additional studies are needed to properly define the role of upfront surgery and identify groups of patients who benefit from this comparably aggressive approach that can be associated with significant or even fatal complications. Therefore, we performed an additional matched-pair study that, in contrast to the previous matched-pair studies, included only patients treated after publication of the Patchell trial. According to its results, the addition of upfront surgery led to a significant improvement of motor function within 3 months after treatment. Surprisingly, this did not result in a significantly higher post-treatment ambulatory rate. The post-treatment ambulatory rate in the surgery plus radiotherapy group was considerably lower than in the Patchell trial [4]. When looking closer at the 32 patients who were not ambulatory following surgery plus radiotherapy, it is noticeable that nine of these patients (28.1%) did not receive radiotherapy at all due to early post-operative mortality and another nine patients (28.1%) did not receive the planned total radiation dose. Of the two patients who experienced an in-field recurrence of MSCC after treatment, one patient did not receive the complete dose of radiotherapy. This might have contributed to the finding that long-term local control of MSCC, represented by freedom from an in-field recurrence and local progression-free survival, was not significantly different in both groups. However, when looking at the 12-month rates of freedom from an in-field recurrence of MSCC, the absolute difference was 13.9% in favor of the combined treatment including upfront surgery.

The fact that 36.7% of the patients in the surgery group did not complete their radiotherapy as planned demonstrates that it is important to carefully select patients who undergo upfront surgery. Patients not completing post-operative radiotherapy had a significantly worse ECOG-PS and were significantly more often not ambulatory than the patients irradiated as planned. Thus, patients with a poor performance status may not benefit from upfront surgery in addition to radiotherapy. This result agrees with the inclusion criteria of the Patchell trial [4]. Moreover, in the present study, patients of the surgery plus radiotherapy group who experienced deterioration of motor deficits during their treatment had a combination of three characteristics (interval from tumor diagnosis to MSCC ≤ 15 months, ECOG-PS of 3–4, visceral metastases) significantly more often than other patients. In particular, these patients did not appear to benefit from upfront surgery.

This finding may also explain why it took 10 years to accrue 101 patients for the Patchell trial, although it was a multi-center study [4]. In the present study, the mortality rate within 30 days following surgery was 17.7%. Rates of surgery-related complications in the Patchell trial were 12% after primary and 40% after salvage treatment, respectively, and the 30-day mortality rate was 6% [4]. In the two previous matched-pair studies, perioperative complications, including wound infections requiring a second surgery, extensive bleeding, pulmonary embolism, and pneumonia, occurred in 11% and 13% of the patients, respectively [5,6]. The 30-day mortality rates were not reported. The risk of death within 30 days further supports the conclusion that patients who may be scheduled for upfront surgery should undergo a careful selection process that choses patients most likely to live longer and complete therapy. Since MSCC is an oncologic emergency situation that often requires treatment within 24 h from the patient’s first presentation, such a process can be quite challenging for the treating physicists. A decision by a multidisciplinary tumor board (MTB), which usually takes place once a week, is too late. Therefore, a close multidisciplinary collaboration outside MTBs between the involved disciplines is also important.

Despite the carefully performed propensity-score matching procedure, this study has several limitations. Most of all, this study is not a randomized trial and, therefore, still bears the risk of a hidden selection bias. Moreover, the Frankel classification is comparably coarse and does not differentiate between being ambulatory without aid and with aid. However, improvement from ambulatory status with aid to becoming ambulatory without aid is likely very important for a patient’s quality of life. Thus, the rates of improvement of motor deficits may be higher in both groups but it remains unknown whether this applies to a similar extent in both groups. The fact that 36.7% of surgically treated patients did not complete radiotherapy as planned might have impaired the results in this group. When interpreting the results of the present study, these limitations need to be considered. Moreover, these results of this study cannot be generalized to the comparably novel approach of separation surgery followed by stereotactic body radiation therapy [18,19,20]. Separation surgery includes epidural decompression and spinal stabilization without gross total or en bloc-resection of the metastasis [21]. In general, instrumented stabilization is performed prior to decompression to avoid the manipulation of hardware across an open spinal canal [21]. In 2013, a retrospective study of 186 patients treated with separation surgery followed by single-fraction radiosurgery or hypo-fractionated SBRT was reported [18]. The cumulative local progression rate at 1 year was 16.4%. Moreover, in a recent phase 2 trial of 33 patients receiving separation surgery plus SBRT with 2 × 12 Gy, the local failure rate at 12 months was 13% [20]. These rates were higher than the 12-month rate of freedom from an in-field recurrence and lower than the 12-month rate of local progression-free survival observed in the surgery plus radiotherapy group of present study. In order to properly define the best combined treatment for MSCC, a randomized trial comparing separation surgery plus SBRT to decompressive surgery plus stabilization followed by volumetric modulated arc therapy using doses per fraction of ≤5 Gy is required. Decompressive surgery should be combined with a stabilization procedure, since this approach results in better outcomes than decompression alone [4,5,6]. Another novel approach includes SBRT followed by surgical stabilization within 24 h for unstable spinal metastases. In a study of 13 patients, this approach appeared safe, and relief of symptoms may occur earlier [22]. However, additional prospective studies and data regarding longer-term control of MSCC are required. One may speculate that upfront radiotherapy could be associated with a higher rate of in-field recurrences than post-operative irradiation in case of intraoperative tumor cell dissemination.

## 4. Materials and Methods

This retrospective study compared surgery followed by radiotherapy and radiotherapy alone for outcomes in patients with MSCC. It was approved by the responsible Ethics Committee (University of Lübeck, reference number: 20-004A). In the radiotherapy alone group, initially data of 461 patients who received radiotherapy with an equivalent dose in 2 Gy fractions (EQD2, [17]) higher than 30 Gy (5 × 5 Gy, 10 × 3 Gy, 14−15 × 2.5 Gy, 20 × 2 Gy) within one of four prospective trials or a validation study were re-analyzed [9,10,11,12,13]. It was aimed to match these patients (propensity score approach) to a cohort of 79 patients who received decompressive surgery and were scheduled for post-operative radiotherapy with similar EQD2 as in the radiotherapy alone group. Patients’ characteristics used for the matching procedure included age at the start of treatment (≤67 vs. ≥68 years, median 67 years), gender, Eastern Cooperative Oncology Group (ECOG) performance score (1.2 vs. 3–4), primary tumor type (breast cancer vs. prostate cancer vs. myeloma/lymphoma vs. lung cancer vs. cancer of unknown primary vs. other tumors), number of vertebrae affected by MSCC (1.2 vs. ≥3), other bone metastases (no vs. yes), visceral metastases (no vs. yes), interval from tumor diagnosis to MSCC (≤15 vs. >15 months), time developing motor deficits (0–7 vs. >7 days), and pre-treatment ambulatory status (not ambulatory vs. ambulatory) (Table 4).

However, most likely due to the fact that upfront surgery is generally performed in selected patients who meet the Patchell criteria [4], the differences of the distributions of the patient characteristics were very pronounced (statistically significant for seven of ten factors) and did not allow proper matching (Table 3). Therefore, the radiotherapy alone group was reduced to the 79 patients who best matched with the surgery plus radiotherapy group. After this procedure, the differences between both groups were considerably less pronounced, and the differences were no longer significant (Table 4).

Both treatment groups (surgery plus radiotherapy vs. radiotherapy alone) were compared with respect to overall effect on motor deficits up to 3 months following treatment (improvement, no further progression, or deterioration), improvement of motor deficits, local progression-free survival (no deterioration of motor deficits during or after treatment), and overall survival. Patients without deterioration of motor deficits during treatment were additionally compared for freedom from an in-field recurrence of MSCC in the treated spinal areas. Motor function was graded with the Frankel grade classification. which includes five grades (A to E, Table 4) [23,24]. Improvement and deterioration of motor deficits were defined as a change of at least one Frankel grade.

The comparisons of both groups with respect to the distributions of the patient characteristics (Table 3) and effect on motor deficits and post-treatment ambulatory status (Table 1) were performed with the Chi square test. For the comparisons with respect to local progression-free survival, overall survival, and freedom from an in-field recurrence of MSCC (Table 2), the Kaplan–Meier method and log-rank test were used. P-values of less than 0.05 were considered significant. Those endpoints that showed a significant difference between both groups were additionally analyzed in a multivariate analysis performed with a logistic regression model plus stepwise regression modeling.

## 5. Conclusions

Given the limitations of the present study, the addition of upfront decompressive surgery to radiotherapy led to significantly better early improvement of motor function and non-significantly better long-term control of MSCC. More than one third of the patients in the surgery plus radiotherapy group did not receive their radiation treatment as planned due to early postoperative death or decreased performance score following surgery. Thus, when selecting a patient for upfront surgery, the individual patient’s prognosis must be considered and weighed against the risk of perioperative complications and the 30-day mortality. Surgery-related complications may impede receiving the complete postoperative radiation treatment as planned. The process of selecting patients for the combined approach may represent a challenge and should be performed by a multidisciplinary team including neurosurgeons and radiation oncologists. In general, upfront surgery should be performed in those patients who would likely benefit in terms of improvement of neurologic deficits and would be able to tolerate the surgery. This applies particularly to patients with a survival prognosis of at least a few months and MSCC from a less radiosensitive tumor, intraspinal bone fragments, sphincter dysfunction, or an unstable spine. Since the patients should likely be able to tolerate the surgery, age, comorbidities, and performance status (ECOG-PS of 0-2) should be considered as well.

## Figures and Tables

**Table 1:** Subgroup analysis in the surgery plus radiotherapy group: Characteristics of the 29 patients who did not complete post-operative radiotherapy (Group A) and 50 patients who completed radiotherapy (Group B).

Characteristic	Group A *n* Patients (%)	Group B *n* Patients (%)	*p*-Value
Age			0.19
≤67 years	16 (55.2)	20 (40.0)	
≥68 years	13 (44.8)	30 (60.0)	
Gender			0.72
Female	11 (37.9)	17 (34.0)	
Male	18 (62.1)	33 (66.0)	
ECOG performance status			**0.001**
1–2	12 (41.4)	39 (78.0)	
3–4	17 (58.6)	11 (22.0)	
Type of primary tumor			0.49
Breast cancer or prostate cancer	5 (17.2)	16 (32.0)	
Myeloma/lymphoma	7 (24.1)	12 (24.0)	
Lung cancer or cancer of unknown primary	10 (34.5)	14 (28.0)	
Other tumors	7 (24.1)	8 (16.0)	
Number of vertebrae affected by MSCC			0.14
1–2	9 (31.0)	24 (48.0)	
≥3	20 (69.0)	26 (52.0)	
Other bone metastases			0.63
No	17 (58.6)	32 (64.0)	
Yes	12 (41.4)	18 (36.0)	
Visceral metastases			0.25
No	16 (55.2)	34 (68.0)	
Yes	13 (44.8)	16 (32.0)	
Interval from tumor diagnosis to MSCC			0.82
≤15 months	21 (72.4)	35 (70.0)	
>15 months	8 (27.6)	15 (30.0)	
Time developing motor deficits			0.39
0–7 days	18 (62.1)	26 (52.0)	
>7 days	11 (37.9)	24 (48.0)	
Ambulatory status			**0.032**
Not ambulatory	20 (69.0)	22 (44.0)	
Ambulatory	9 (31.0)	28 (56.0)	

The *p*-values were obtained with the Chi-square test. ECOG: Eastern Cooperative Oncology Group; MSCC: metastatic spinal cord compression; bold values: significant *p*-values.

**Table 2:** Comparison of surgery plus radiotherapy and radiotherapy alone with respect to the effect on motor deficits and post-treatment ambulatory status.

Endpoint	Surgery Plus Radiotherapy *n* Patients (%)	Radiotherapy Alone *n* Patients (%)	*p*-Value
Overall effect on motor deficits			**0.009**
Improvement	31 (39.2)	17 (21.5)	
No further progression	36 (45.6)	55 (69.6)	
Deterioration	12 (15.2)	7 (8.9)	
Improvement of motor deficits			**0.015**
No	48 (60.8)	62 (78.5)	
Yes	31 (39.2)	17 (21.5)	
Post-treatment ambulatory status			0.32
Not Ambulatory	32 (40.5)	26 (32.9)	
Ambulatory	47 (59.5)	53 (67.1)	

Bold values: significant *p*-values.

**Table 3:** Comparison of surgery plus radiotherapy and radiotherapy alone with respect to local progression-free survival, overall survival, and freedom from an in-field recurrence of MSCC.

Endpoint	Surgery Plus Radiotherapy	Radiotherapy Alone	*p*-Value
Local progression-free survival			0.47
At 6 months	80.3%	88.6%	
At 12 months	72.6%	68.8%	
Overall survival			0.51
At 6 months	58.1%	52.0%	
At 12 months	42.1%	32.5%	
Mortality within 30 days following surgery or radiotherapy alone	15.2%	12.7%	0.65
Freedom from in-field recurrence of MSCC *			0.58
At 6 months	94.4%	98.2%	
At 12 months	90.1%	76.2%	

MSCC: metastatic spinal cord compression; * analysis included only patients without deterioration of motor deficits during treatment.

**Table 4:** Distribution of the patient characteristics used for propensity score matching in both treatment groups. In the radiotherapy alone group, the distributions for the entire cohort of 461 patients (initial matching) and the final cohort of 79 patients (final matching) are shown.

	OP + RT *n* Patients (%)	RT (All) *p*-Value *n* Patients (%)	RT (Matched Subgroup) *p*-Value *n* Patients (%)
Age		*p* = 0.23	*p* = 0.75
≤67 years	36 (45.6)	244 (52.9)	38 (48.1)
≥68 years	43 (54.4)	217 (47.1)	41 (51.9)
Gender		*p* = 0.13	*p* = 0.74
Female	28 (35.4)	205 (44.5)	30 (38.0)
Male	51 (64.6)	256 (55.5)	49 (62.0)
ECOG performance status		***p* < 0.001**	*p* = 0.26
1–2	51 (64.6)	197 (42.7)	44 (55.7)
3–4	28 (35.4)	264 (57.3)	35 (44.3)
Type of primary tumor		***p* < 0.001**	*p* = 0.60
Breast cancer	8 (10.1)	121 (26.3)	13 (16.5)
Prostate cancer	13 (16.5)	79 (17.1)	14 (17.7)
Myeloma/lymphoma	19 (24.1)	43 (9.3)	11 (13.9)
Lung cancer	18 (22.8)	106 (23.0)	18 (22.8)
Cancer of unknown primary	6 (7.6)	23 (5.0)	8 (10.1)
Other tumors	15 (19.0)	89 (19.3)	15 (19.0)
Number of vertebrae affected by MSCC		*p* = 0.82	*p* = 0.87
1–2	33 (41.8)	199 (43.2)	34 (43.0)
≥3	46 (58.2)	262 (56.8)	45 (57.0)
Other bone metastases		***p* < 0.001**	*p* = 0.63
No	49 (62.0)	80 (17.4)	46 (58.2)
Yes	30 (38.0)	380 (82.6)	33 (41.8)
Visceral metastases		***p* = 0.002**	*p* = 0.15
No	50 (63.3)	205 (44.5)	41 (51.9)
Yes	29 (37.7)	256 (55.5)	38 (48.1)
Interval from tumor diagnosis to MSCC		***p* = 0.033**	*p* = 0.39
≤15 months	56 (70.9)	268 (58.1)	51 (64.6)
>15 months	23 (29.1)	193 (41.9)	28 (35.4)
Time developing motor deficits		***p* < 0.001**	*p* = 0.26
0–7 days	44 (55.7)	146 (31.7)	37 (46.8)
>7 days	35 (44.3)	315 (68.3)	42 (53.2)
Ambulatory status		***p* = 0.002**	*p* = 0.52
Not ambulatory	42 (53.2)	160 (34.7)	38 (48.1)
Ambulatory	37 (46.8)	301 (65.3)	41 (51.9)

The *p*-values are given for comparisons with surgery plus radiotherapy and were obtained with the Chi-square test. ECOG: Eastern Cooperative Oncology Group; MSCC: metastatic spinal cord compression; bold values: significant *p*-values.

## Data Availability

Data of three prospective trials (NCT03070431, NCT02189473, and NCT04043156) are available at clinicaltrials.gov. Otherwise, the data analyzed for this paper cannot be shared due to data protection regulations. According to the ethics committee, only evaluation of anonymized data is allowed for this study.

## References

[1] Prasad D., Schiff D. (2005). Malignant spinal cord compression. Lancet Oncol..

[2] Rades D., Abrahm J.L. (2010). The role of radiotherapy for metastatic epidural spinal cord compression. Nat. Rev. Clin. Oncol..

[3] Lawton A.J., Lee K.A., Cheville A.L., Ferrone M.L., Rades D., Balboni T.A., Abrahm J.L. (2019). Assessment and management of patients with metastatic spinal cord compression: A multidisciplinary review. J. Clin. Oncol..

[4] Patchell R., Tibbs P.A., Regine W.F., Payne R., Saris S., Kryscio R.J., Mohiuddin M., Young B. (2005). Direct decompressive surgical resection in the treatment of spinal cord compression caused by metastatic cancer: A randomised trial. Lancet.

[5] Rades D., Huttenlocher S., Dunst J., Bajrovic A., Karstens J.H., Rudat V., Schild S.E. (2010). Matched pair analysis comparing surgery followed by radiotherapy and radiotherapy alone for metastatic spinal cord compression. J. Clin. Oncol..

[6] Rades D., Huttenlocher S., Bajrovic A., Karstens J.H., Adamietz I.A., Kazic N., Rudat V., Schild S.E. (2011). Surgery followed by radiotherapy versus radiotherapy alone for metastatic spinal cord compression from unfavorable tumors. Int. J. Radiat. Oncol. Biol. Phys..

[7] Maseda M., Uei H., Nakahashi M., Sawada H., Tokuhashi Y. (2019). Neurological outcome of treatment for patients with impending paralysis due to epidural spinal cord compression by metastatic spinal tumor. J. Orthop. Surg. Res..

[8] Barzilai O., Robin A.M., O’Toole J.E., Laufer I. (2020). Minimally invasive surgery strategies: Changing the time of spine tumors. Neurosurg. Clin. N. Am..

[9] Rades D., Cacicedo J., Conde-Moreno A.J., Segedin B., But-Hadzic J., Groselj B., Kevlishvili G., Lomidze D., Ciervide-Jurio R., Rubio C. (2020). Precision radiation therapy for metastatic spinal cord compression: Final results of the PRE-MODE Trial. Int. J. Radiat. Oncol. Biol. Phys..

[10] Rades D., Hansen O., Jensen L.H., Dziggel L., Staackmann C., Doemer C., Cacicedo J., Conde-Moreno A.J., Segedin B., Ciervide-Jurio R. (2019). Radiotherapy for metastatic spinal cord compression with increased radiation doses (RAMSES-01): A prospective multicenter study. BMC Cancer.

[11] Rades D., Lange M., Veninga T., Stalpers L.J., Bajrovic A., Adamietz I.A., Rudat V., Schild S.E. (2011). Final results of a prospective study comparing the local control of short-course and long-course radiotherapy for metastatic spinal cord compression. Int. J. Radiat. Oncol. Biol. Phys..

[12] Rades D., Šegedin B., Conde-Moreno A.J., Garcia R., Perpar A., Metz M., Badakhshi H., Schreiber A., Nitsche M., Hipp P. (2016). Radiotherapy with 4 Gy × 5 versus 3 Gy × 10 for metastatic epidural spinal cord compression: Final results of the SCORE-2 trial (ARO 2009/01). J. Clin. Oncol..

[13] Rades D., Douglas S., Veninga T., Stalpers L.J.A., Hoskin P.J., Bajrovic A., Adamietz I.A., Basic H., Dunst J., Schild S.E. (2010). Validation and simplification of a score predicting survival in patients irradiated for metastatic spinal cord compression. Cancer.

[14] Joiner M.C., Van der Kogel A.J., Steel G.G. (1997). The linear-quadratic approach to fractionation and calculation of isoeffect relationships. Basic Clinical Radiobiology.

[15] van den Bent M.J. (2005). Surgical resection improves outcome in metastatic epidural spinal cord compression. Lancet.

[16] Kunkler I. (2006). Surgical resection in metastatic spinal cord compression. Lancet.

[17] Knisely J., Strugar J. (2006). Can decompressive surgery improve outcome in patients with metastatic epidural spinal-cord compression?. Nat. Clin. Pract. Oncol..

[18] Laufer I., Iorgulescu J.B., Chapman T., Lis E., Shi W., Zhang Z., Cox B.W., Yamada Y., Bilsky M.H. (2013). Local disease control for spinal metastases following “separation surgery” and adjuvant hypofractionated or high-dose single-fraction stereotactic radiosurgery: Outcome analysis in 186 patients. J. Neurosurg. Spine.

[19] Turel M.K., Kerolus M.G., O’Toole J.E. (2017). Minimally invasive “separation surgery” plus adjuvant stereotactic radiotherapy in the management of spinal epidural metastases. J. Craniovertebr. Junction Spine.

[20] Ito K., Sugita S., Nakajima Y., Furuya T., Hiroaki O., Hayakawa S., Hozumi T., Saito M., Karasawa K. (2022). Phase 2 clinical trial of separation surgery followed by stereotactic body radiation therapy for metastatic epidural spinal cord compression. Int. J. Radiat. Oncol. Biol. Phys..

[21] Moussazadeh N., Laufer I., Yamada Y., Bilsky M.H. (2014). Separation surgery for spinal metastases: Effect of spinal radiosurgery on surgical treatment goals. Cancer Control.

[22] Versteeg A.L., van der Velden J.M., Hes J., Eppinga W., Kasperts N., Verkooijen H.M., Oner F.C., Seravalli E., Verlaan J.J. (2018). Stereotactic radiotherapy followed by surgical stabilization within 24 h for unstable spinal metastases; A stage I/II a study according to the IDEAL Framework. Front. Oncol..

[23] Frankel H.L., Hancock D.O., Hyslop G., Melzak J., Michaelis L.S., Ungar G.H., Vernon J.D.S., Walsh J.J. (1969). The value of postural reduction in the initial management of closed injuries of the spine with paraplegia and tetraplegia. Spinal Cord..

[24] Kirshblum S., Botticello A., Benedetto J., Donovan J., Marino R., Hsieh S., Wagaman N. (2020). A comparison of diagnostic stability of the ASIA Impairment Scale versus Frankel Classification Systems for traumatic spinal cord injury. Arch. Phys. Med. Rehabil..

